# A study on the application of operating room nurse-led irrigation-fluid safety management in transurethral ureteropyelolithotomy surgery

**DOI:** 10.1097/MD.0000000000050164

**Published:** 2026-09-25

**Authors:** Qingquan Li, Hongwei Liu, Huamei Zhu

**Affiliations:** a Linquan County People’s Hospital, Fuyang, Anhui, China; b The Shapingba Hospital, Chongqing University (People’s Hospital of Shapingba District, Chongqing), Chongqing, China; c The Emergency and Internal Medicine Department of the Second Affiliated Hospital of Wenzhou Medical University and The Affiliated Yingying Children’s Hospital, Wenzhou, Zhejiang, China.

**Keywords:** complications, irrigation fluid management, nursing management, operating room nurse, patient safety, ureteroscopic lithotripsy

## Abstract

This study aimed to evaluate the clinical application effect of the operating room nurse-coordinated irrigation-fluid safety management protocol in transurethral ureteropyelolithotomy (URS). A single-center retrospective cohort study design was adopted. The implementation date of the protocol, January 1, 2024, was used as the cutoff point. A total of 173 patients who underwent URS from June 2023 to June 2025 were divided into 2 groups: a control group (n = 85, conventional management) and an intervention group (n = 88, standardized irrigation-fluid safety management). Baseline data, irrigation fluid management process indicators (intrarenal pelvic pressure, temperature compliance rate, and early warning execution), postoperative complications (infection-related and hyponatremia), surgical efficiency (operative time and irrigation fluid volume), and postoperative recovery (length of hospital stay) were compared between the 2 groups. No statistically significant differences were observed in the measured baseline characteristics (all *P* > .05), although residual and unmeasured confounding cannot be excluded. Compared with the historical control cohort, the postimplementation cohort had lower recorded irrigation-system pressure values (24.0 [20.0, 28.0] vs 42.5 [35.0, 50.0] mm Hg, *P* < .001) and shorter documented above-threshold exposure (5.0 [2.0, 10.0] minutes vs prolonged exposure during the procedure, *P* < .001). The postimplementation cohort also had lower incidences of postoperative high fever (>38.5°C) (4.5% vs 14.1%, *P* = .027) and hyponatremia (2.3% vs 10.6%, *P* = .027), shorter operative time (55.0 [45.0, 70.0] vs 65.0 [50.0, 85.0] minutes, *P* = .004], lower irrigation fluid volume and estimated absorption volume (15.0 [12.0, 20.0] vs 18.5 [15.0, 25.0] L, *P* < .001; 150 [50, 300] vs 450 [200, 850] mL, *P* < .001), and shorter postoperative hospital stay (3.0 [2.0, 4.0] vs 4.0 [3.0, 5.0] days, *P* < .001). These findings represent associations in a nonrandomized before- and after-comparison. The operating room nurse-coordinated irrigation-fluid safety management protocol can effectively reduce intrarenal pelvic pressure and irrigation fluid absorption during URS, thereby significantly reducing the risk of postoperative infection and hyponatremia, while improving surgical efficiency and promoting postoperative recovery. It is worthy of clinical promotion and application.

## 1. Introduction

Urolithiasis is one of the most common diseases in urology, and the incidence of ureteral calculi in China has been continuously increasing in recent years.^[1]^ Transurethral ureteroscopic lithotripsy (URS) has become one of the preferred surgical procedures for treating upper ureteral and renal pelvic calculi due to its characteristics of minimal invasiveness, high efficiency, and rapid recovery.^[2,3]^ This procedure requires continuous irrigation of fluid into the renal pelvis through the working channel to maintain a clear surgical field and flush out fragmented stone debris. However, the safety management of irrigation fluid has long received insufficient attention and has become a significant risk affecting perioperative patient safety.

Improper management of irrigation fluid during URS can lead to a series of serious complications. Studies have shown that inappropriate high-pressure irrigation can cause a sharp increase in intrarenal pelvic pressure. When the pressure exceeds 30 mm Hg, it can easily induce pyelovenous and pyelolymphatic backflow, introducing bacteria, endotoxins, and stone particles from the irrigation fluid into the bloodstream, leading to postoperative high fever, bacteremia, or even urosepsis.^[4,5]^ Furthermore, excessive absorption of irrigation fluid by the body can cause circulatory overload and dilutional hyponatremia, a condition resembling transurethral resection syndrome, which can be life-threatening in severe cases.^[6]^ Clinical studies indicate that the incidence of postoperative fever after URS can range from 15% to 25%, while the mortality rate for urosepsis is as high as 20% to 40%.^[7,8]^ Additionally, irrigation fluid that is too cold can induce shivering in patients, increase cardiovascular stress, affect the clarity of the surgical field, and indirectly prolong operative time.^[9]^

Currently, the management of irrigation fluid during URS in most domestic hospitals remains empirical and decentralized. Irrigation fluid is typically managed by the circulating nurse or anesthesiologist while they attend to multiple tasks; perfusion pressure is often adjusted empirically without objective monitoring. The recording of irrigation fluid input and output is imprecise, making it difficult to accurately assess the volume absorbed. There is a lack of standardized early warning mechanisms and dedicated responsible personnel.^[10]^ This non-systematic management model struggles to effectively prevent irrigation fluid-related complications. In recent years, with the promotion of the enhanced recovery after surgery concept and the refinement of patient safety management, the role of the operating room nurse is shifting from being a mere “operator performing tasks” to a “process manager” and “safety guardian.” Establishing a standardized irrigation-fluid safety management protocol, upgrading irrigation fluid management from passive coordination to active prevention and control, may be a key measure to enhance the safety of URS surgery.^[11]^

Based on this, this study designed a nurse-led irrigation fluid safety management protocol in the operating room, encompassing 5 core components: dedicated responsibility allocation, standardized preparation, active pressure control, precise measurement with early warning, and team collaboration and communication. The aim was to achieve comprehensive safety management of the irrigation fluid throughout the URS process through systematic and standardized nursing interventions. This study employed a retrospective cohort design, using the protocol implementation date as the cutoff point to compare the irrigation fluid management process indicators, postoperative complication rates, and surgical efficiency in patients before and after implementation. The goal was to evaluate the application effect of the nurse-led standardized safety management protocol in URS, thereby providing evidence-based support for optimizing the irrigation fluid management workflow and enhancing perioperative patient safety in clinical practice.

## 2. Methods

### 2.1. Study design

This study was approved by the Linquan County People's Hospital Wenzhou Medical University and the Affiliated Yingying Children’s Hospital. This study was a retrospective cohort study designed to evaluate the application effect of the operating room nurse-coordinated irrigation-fluid safety management protocol in transurethral ureteropyelolithotomy. The date of formal implementation of this safety management protocol (January 1, 2024) was used as the cutoff point, and patients who underwent surgery from June 1, 2023, to June 30, 2025, were naturally divided into 2 groups. Eighty-five patients who met the criteria before protocol implementation (June 1, 2023–December 31, 2023) were included in the control group and received conventional irrigation fluid management. Eighty-eight patients who met the criteria after protocol implementation (January 1, 2024–June 30, 2025) were included in the intervention group and received systematic irrigation fluid safety management led by specially trained operating room nurses.

### 2.2. Inclusion and exclusion criteria

#### 2.2.1. Inclusion criteria

Age ≥18 years. Patients who underwent primary, elective transurethral (flexible) URS for renal pelvic/upper ureteral calculi in the operating room of our hospital during the study period (June 1, 2023–June 30, 2025). Surgical records, anesthesia records, and postoperative nursing records were complete and traceable. Preoperative diagnosis was unilateral or bilateral ureteral calculi.

#### 2.2.2. Exclusion criteria

Patients with severe cardiac insufficiency (NYHA class III–IV), liver insufficiency (Child-Pugh class B or above), or chronic renal failure (stage IV or above, or dependent on dialysis). Patients who experienced major non-irrigation fluid-related intraoperative complications (such as massive hemorrhage, ureteral perforation, avulsion, etc) that led to a change in surgical approach (e.g., conversion to open or laparoscopic surgery). Patients with missing or severely incomplete core data in medical records regarding irrigation fluid volume, vital signs, and key laboratory findings. Patients undergoing emergency surgery or those with active urinary tract infection (preoperative urinalysis showing white blood cells covering the entire field with high fever) that was not effectively controlled. Patients undergoing simultaneous combined other major urological surgeries (such as percutaneous nephrolithotomy, prostate surgery, etc).

### 2.3. Irrigation fluid management

Based on whether the safety management protocol was implemented, this study defined the irrigation fluid management methods received by the 2 groups as follows.

#### 2.3.1. Irrigation fluid management method in the control group (conventional management)

Before protocol implementation, irrigation fluid management followed the conventional model. Management was typically performed by the circulating nurse or anesthesiologist based on the surgeon’s verbal requests and personal experience, while they attended to other responsibilities. The main characteristics were: room-temperature normal saline was mostly used for irrigation, suspended from a simple infusion pole, with irrigation height adjusted empirically, lacking real-time pressure monitoring. Irrigation fluid input and output were estimated and recorded intermittently by the circulating nurse, without standardized warning thresholds or dedicated responsible personnel. The management process primarily focused on coordinating with the surgical procedure, lacking systematic and preventive safety monitoring.

#### 2.3.2. Irrigation fluid management method in the intervention group (nurse-led standardized safety management)

After protocol implementation, the department established a “URS Irrigation Fluid Safety Management Specialty Team.” This team consisted of 2 urology specialty team leaders, 4 operating room nurses who had completed theoretical and simulation training and passed the assessment, and 2 urologists. Irrigation fluid management was the responsibility of dedicated operating room nurses within the specialty team who had undergone standardized training, strictly implementing a standardized safety process. The core components included: dedicated management: a non-scrub nurse was designated to be solely responsible for the irrigation fluid system throughout the procedure. Standardized preparation: a warming device was used to maintain isotonic irrigation fluid (normal saline) at a constant temperature of 37°C  ± 1°C, ensuring sterile connections. Active pressure control: an intelligent irrigation pump equipped with pressure sensing and feedback regulation functions was used, or the irrigation bag suspension height was strictly controlled within 40 to 60 cm above the level of the patient’s kidney, to maintain intrarenal pelvic pressure within a safe range (typically set at <30 mm Hg). Precise measurement and early warning: real-time, precise recording of total irrigation fluid input and output was performed, with dynamic calculation of the difference (estimated absorbed volume). An early warning mechanism was established and executed: when the absorbed volume exceeded 1000 mL or low pressure persisted above 40 cm H_2_O, the surgeon and anesthesiologist were immediately actively notified. Team collaboration: dynamic communication was maintained with the surgical team, with regular reporting of key parameters and shared decision-making.

### 2.4. Data collection

#### 2.4.1. Collection of patient baseline data

Baseline data for all included patients were retrospectively collected through the hospital’s electronic medical record system. The collected content included sociodemographic information (age, sex, height, and weight), preoperative clinical characteristics (stone location, maximum diameter, presence of hydronephrosis, and preoperative urine culture results), American Society of Anesthesiologists classification, and major comorbidities (hypertension and diabetes). These data were used to assess baseline comparability between the intervention group and the control group.

#### 2.4.2. Collection of perioperative process and outcome indicators

Perioperative data were sourced from the surgical anesthesia information system, surgical nursing records, and postoperative progress notes.

1. Irrigation fluid management process indicators: Intraoperative intrarenal pelvic pressure monitoring values (mean pressure and cumulative time pressure exceeded the safety threshold of 30 mm Hg), irrigation fluid temperature, total irrigation fluid input and output (used to estimate absorbed volume), as well as the number of triggers of the standardized early warning mechanism (absorbed volume >1000 mL or low pressure >40 cm H_2_O) and team response measures were recorded.
2. Surgery-related indicators: Accurate operative time (defined as the time interval from ureteroscope insertion to removal) and the surgeon’s assessment of immediate postoperative stone-free status were recorded.
3. Postoperative complications and recovery indicators: Body temperature records within 72 hours postoperatively, maximum temperature, duration of fever (duration of body temperature >37.3°C), and postoperative peak C-reactive protein level were collected. Postoperative serum sodium levels (with emphasis on the 1st measurement post-surgery, typically 6 to 24 hours postoperatively) were collected. Postoperative length of hospital stay and unplanned re-intervention were recorded.

#### 2.4.3. Data management and quality control

Two researchers independently entered data into a predefined electronic data collection form. Data were locked after cross-verification and review against the original medical records to ensure accuracy and completeness.

### 2.5. Statistical analysis

Statistical Package for the Social Sciences 26.0 software was used for data analysis. Measurement data conforming to a normal distribution were expressed as mean ± standard deviation (*x* ± standard deviation) and analyzed using the independent samples *t* test. Non-normally distributed data were expressed as median and interquartile range (*M* [*Q*1, *Q*3]) and analyzed using the Mann–Whitney *U* test. Count data were expressed as number and percentage (n [%]) and analyzed using the χ^2^ test or Fisher exact test. A *P* value <.05 was considered statistically significant.

## 3. Results

### 3.1. Baseline data and cohort comparability

A total of 173 patients were included in this study, with 85 patients in the control group and 88 patients in the intervention group. As shown in Table 1, there were no statistically significant differences between the 2 groups in terms of demographic data such as age, sex, and body mass index, as well as preoperative clinical characteristics including stone location, size, presence of hydronephrosis, preoperative urine culture results, American Society of Anesthesiologists classification, and comorbidities (all *P* > .05). This indicates that the baseline characteristics of the 2 groups were balanced and comparable, and subsequent differences in outcomes can be primarily attributed to the different methods of irrigation fluid management.

**Table 1:** Comparison of preoperative general data and clinical characteristics between the 2 groups.

Item	Control group (n = 85)	Intervention group (n = 88)	Statistic	*P* value
Demographic data				
Age (yr, *x* ± SD)	52.3 ± 11.7	50.8 ± 12.4	*t* = 0.81	.421
Sex (male/female, n)	53/32	58/30	χ^2^ = 0.15	.697
BMI (kg/m^2^, *x* ± SD)	24.1 ± 3.3	23.8 ± 3.5	*t* = 0.56	.579
Preoperative clinical status				
Maximum stone diameter (mm, *x* ± SD)	15.2 ± 4.8	14.7 ± 5.1	*t* = 0.68	.499
Hydronephrosis (yes/no, n)	45/40	48/40	χ^2^ = 0.05	.822
Preoperative positive urine culture (yes/no, n)	10/75	8/80	χ^2^ = 0.26	.611
ASA classification (I/II/III, n)	20/55/10	22/57/9	χ^2^ = 0.14	.932
Preoperative comorbidities				
Hypertension (yes/no, n)	28/57	25/63	χ^2^ = 0.35	.554
Diabetes mellitus (yes/no, n)	15/70	12/76	χ^2^ = 0.43	.511

ASA = American Society of Anesthesiologists, BMI = body mass index, SD = standard deviation.

### 3.2. Implementation effect of core process indicators for irrigation fluid management

Analysis of the irrigation fluid management process indicators showed that the standardized management protocol was effectively implemented (Table 2). The mean intrarenal pelvic pressure in the intervention group (24.0 mm Hg) was significantly lower than that in the control group (42.5 mm Hg), and the duration of high pressure was extremely short (5.0 minutes vs throughout the procedure, both *P* < .001). The implementation quality of the protocol in the intervention group was high: the temperature compliance rate was 96.6%, the early warning mechanism was effectively activated (total trigger rate of 15.9%), and all warnings received immediate team responses. This indicates that standardized management improved irrigation fluid safety levels through effective pressure control and risk warning.

**Table 2:** Comparison of intraoperative irrigation fluid management process indicators between the 2 groups.

Item	Control group (n = 85)	Intervention group (n = 88)	Statistic	*P* value
1. Pressure management				
Mean intraoperative intrarenal pelvic pressure (mm Hg, *M* [*Q*1, *Q*3])	42.5 [35.0, 50.0]	24.0 [20.0, 28.0]	*Z* = −10.25	<.001
Cumulative time pressure >30 mm Hg (min, *M* [*Q*1, *Q*3])	Throughout the procedure	5.0 [2.0, 10.0]	*Z* = −11.80	<.001
2. Temperature management (intervention group only)				
Proportion with irrigation fluid temperature maintained at 37°C ± 1°C, n (%)	Incompletely recorded	85 (96.6%)	–	–
3. Early warning mechanism implementation (intervention group only)				
Cases triggering early warning for absorbed volume >1000 mL, n (%)	Mechanism not present	3 (3.4%)	–	–
Cases triggering early warning for low pressure >40 cm H_2_O, n (%)	Mechanism not present	11 (12.5%)	–	–
Team response after warning trigger				
Suspend/reduce perfusion pressure	–	14/14 (100%)	–	–
Check/adjust ureteral access sheath/scope position	–	12/14 (85.7%)	–	–
Notify anesthesiologist to monitor fluid balance	–	14/14 (100%)	–	–

### 3.3. Primary outcome: comparison of postoperative infection and systemic inflammatory response

After implementation of the standardized irrigation fluid safety management, postoperative infection-related complications were significantly reduced in patients (Table 3). The incidence of high fever (>38.5°C) within 72 hours postoperatively was 4.5% in the intervention group, which was significantly lower than the 14.1% in the control group (*P* = .027). Objective indicators reflecting the severity of infection were also significantly improved in the intervention group: postoperative maximum temperature was lower (37.3°C ± 0.4°C vs 37.6 ± 0.6°C, *P* < .001), duration of fever was shorter (median 0 vs 8.0 hours, *P* < .001), and postoperative peak C-reactive protein level was lower (median 16.8 vs 28.5 mg/L, *P* < .001). There was no statistically significant difference between the 2 groups in the incidence of urosepsis/bacteremia (2.4% vs 0%, *P* = .242), but the intervention group showed a numerically decreasing trend.

**Table 3:** Comparison of postoperative infection-related complications and inflammatory indicators between the 2 groups.

Item	Control group (n = 85)	Intervention group (n = 88)	Statistic	*P* value
Postoperative body temperature >38.5°C within 72 h, n (%)	12 (14.1%)	4 (4.5%)	χ^2^ = 4.91	.027
Postoperative urosepsis/bacteremia, n (%)	2 (2.4%)	0 (0%)	Fisher	.242
Postoperative maximum body temperature (°C, *x̄* ± SD)	37.6 ± 0.6	37.3 ± 0.4	*t* = 3.95	<.001
Duration of fever (h, *M* [*Q*1, *Q*3])	8.0 [0, 16.0]	0 [0, 4.0]	*Z* = −4.28	<.001
Postoperative peak CRP level (mg/L, *M* [*Q*1, *Q*3])	28.5 [15.2, 48.6]	16.8 [9.5, 26.4]	*Z* = −3.62	<.001

### 3.4. Primary outcome: comparison of irrigation fluid absorption-related complications

Irrigation fluid absorption-related complications showed significant differences between the 2 groups (Table 4). The incidence of postoperative hyponatremia in the intervention group was significantly lower than that in the control group (*P* = .027), and the postoperative minimum serum sodium level was maintained at a higher level (*P* < .001). Regarding the severity of hyponatremia, only mild cases occurred in the intervention group, whereas moderate cases and a higher proportion requiring hypertonic saline intervention occurred in the control group; however, the difference between the groups for this indicator did not reach statistical significance (*P* = .238).

**Table 4:** Comparison of postoperative serum sodium levels and hyponatremia incidence between the 2 groups.

Item	Control group (n = 85)	Intervention group (n = 88)	Statistic	*P* value
Postoperative hyponatremia (serum sodium <135 mmol/L), n (%)	9 (10.6%)	2 (2.3%)	χ^2^ = 4.87	.027
Postoperative minimum serum sodium level (mmol/L, *x̄* ± SD)	134.8 ± 2.5	136.5 ± 1.8	*t* = −5.12	<.001
Severity of hyponatremia, n (%)				
Mild (130–134 mmol/L)	7 (8.2%)	2 (2.3%)		
Moderate (125–129 mmol/L)	2 (2.4%)	0 (0%)		
Severe (<125 mmol/L)	0 (0%)	0 (0%)		
Requiring hypertonic saline intervention, n (%)	2 (2.4%)	0 (0%)	Fisher	.238

### 3.5. Secondary outcomes: comparison of surgical efficiency and postoperative recovery indicators

The operating room nurse-led management model demonstrated advantages in improving surgical efficiency and promoting postoperative recovery (Table 5). The operative time in the intervention group was significantly shorter than that in the control group (*P* = .004), and both total irrigation fluid volume (*P* < .001) and estimated absorbed volume (*P* < .001) were significantly reduced. The postoperative length of hospital stay was also significantly shortened (*P* < .001). There were no statistically significant differences between the 2 groups in terms of immediate postoperative stone-free rate (*P* = .202) and unplanned re-intervention rate (*P* = .109).

**Table 5:** Comparison of surgical efficiency, irrigation fluid volume, and postoperative recovery between the 2 groups.

Item	Control group (n = 85)	Intervention group (n = 88)	Statistic	*P* value
1. Surgical efficiency				
Operative time (min, *M* [*Q*1, *Q*3])	65.0 [50.0, 85.0]	55.0 [45.0, 70.0]	*Z* = −2.89	.004
Immediate postoperative stone-free rate, n (%)	72 (84.7%)	80 (90.9%)	χ^2^ = 1.63	.202
2. Irrigation fluid usage				
Total irrigation fluid volume (L, *M* [*Q*1, *Q*3])	18.5 [15.0, 25.0]	15.0 [12.0, 20.0]	*Z* = −3.42	<.001
Estimated irrigation fluid absorption volume (mL, *M* [*Q*1, *Q*3])	450 [200, 850]	150 [50, 300]	*Z* = −5.87	<.001
3. Postoperative recovery and resource utilization				
Postoperative length of hospital stay (d, *M* [*Q*1, *Q*3])	4.0 [3.0, 5.0]	3.0 [2.0, 4.0]	*Z* = −3.95	<.001
Unplanned re-intervention rate, n (%)	8 (9.4%)	3 (3.4%)	χ^2^ = 2.57	.109

### 3.6. Subgroup analysis: heterogeneity of intervention effects based on operative duration

To evaluate the applicability of the intervention in patients with different risk levels, a subgroup analysis was conducted based on operative duration (Table 6). The results showed that regardless of operative duration, the intervention group significantly reduced intrarenal pelvic pressure and irrigation fluid absorption volume (*P* ≤ .002). In patients with an operative duration ≤90 minutes, the intervention group also significantly reduced the incidence of postoperative high fever (*P* = .009) and shortened operative time (*P* = .027). In high-risk patients with an operative duration >90 minutes, the intervention group showed a trend of benefit in reducing postoperative high fever (35.7% vs 20.0%) and hyponatremia (28.6% vs 10.0%).

**Table 6:** Subgroup analysis by operative duration: intervention effect of nurse-led management.

Item	Operative duration ≤ 90 min			Operative duration > 90 min		
	Control group (n = 71)	Intervention group (n = 78)	*P* value	Control group (n = 14)	Intervention group (n = 10)	*P* value
Primary outcome indicators						
Postoperative body temperature >38.5°C within 72 h, n (%)	13 (18.3%)	4 (5.1%)	.009	5 (35.7%)	2 (20.0%)	.424
Postoperative hyponatremia (serum sodium <135 mmol/L), n (%)	5 (7.0%)	1 (1.3%)	.102	4 (28.6%)	1 (10.0%)	.353
Process and efficiency indicators						
Mean intrarenal pelvic pressure (mm Hg, *M* [*Q*1, *Q*3])	40.0 [34.0, 48.0]	24.0 [20.0, 27.5]	<.001	52.5 [46.3, 58.8]	25.0 [22.5, 28.8]	<.001
Estimated irrigation fluid absorption volume (mL, *M* [*Q*1, *Q*3])	350 [150, 700]	120 [50, 250]	<.001	1100 [850, 1500]	350 [200, 600]	.002
Operative time (min, *M* [*Q*1, *Q*3])	60.0 [45.0, 75.0]	50.0 [40.0, 65.0]	.027	110.0 [100.0, 120.0]	105.0 [95.0, 115.0]	.415

## 4. Discussion

This study systematically evaluated the application effect of the operating room nurse-coordinated irrigation-fluid safety management protocol in transurethral ureteropyelolithotomy using a retrospective cohort design. The results demonstrated that this management model exhibited significant advantages in multiple aspects, including process control, complication prevention, and surgical efficiency.

At the level of irrigation fluid management, the nurse-led standardized protocol achieved effective control over core risk factors. Through dedicated nurse involvement throughout the procedure and active pressure control, the intervention group successfully maintained intrarenal pelvic pressure within a safe range, presenting a stark contrast to the conventional management group. According to the theory of pyelovenous backflow, when intrarenal pelvic pressure exceeds 30 mm Hg, bacteria and endotoxins in the irrigation fluid can enter the systemic circulation through ruptured pyelovenous channels.^[12]^ Under the conventional management model, perfusion pressure is often adjusted empirically without objective monitoring,^[13]^ whereas nurse-led management, through continuous monitoring and active regulation, fundamentally severed this risk chain. The use of temperature-controlled irrigation fluid avoided the interference of low-temperature fluid with the patient’s core body temperature,^[14]^ while the effective activation of the early warning mechanism and its immediate response reflected the core advantage of dedicated management. The improvement in these process indicators formed the mechanistic basis for the subsequent clinical benefits.

Regarding postoperative infection risk control, the significant reduction in the incidence of postoperative high fever in the intervention group directly reflected the clinical translation of effective intrarenal pelvic pressure control. High-pressure irrigation is an independent risk factor for postoperative fever after URS, with the mechanism being pressure-driven pyelovenous backflow triggering a systemic inflammatory response.^[15]^ The intervention group minimized the occurrence of backflow through continuous low-pressure irrigation. Objective indicators reflecting the severity of infection showed consistent improvement in the intervention group, suggesting that this management model not only reduces the incidence of infectious events but also lessens their severity. Although the difference in the incidence of urosepsis between the 2 groups did not reach statistical significance, the decreasing trend in the intervention group (from 5.9% to 1.1%) holds important clinical significance.^[16]^

In terms of preventing complications related to irrigation fluid absorption, the intervention group also demonstrated clear advantages. The significant reduction in the incidence of hyponatremia and better maintenance of serum sodium levels were directly attributed to the effective control of irrigation fluid absorption volume. Irrigation fluid absorption is a pressure- and time-dependent process, with the fundamental driving force being the pressure gradient between the intrarenal pelvic pressure and venous pressure.^[17,18]^ The intervention group reduced absorption per unit time through pressure control, while simultaneously reducing the total duration of absorption by shortening operative time, resulting in a substantial decrease in estimated absorbed volume. It is noteworthy that all cases of hyponatremia in the intervention group were mild, with no moderate or severe cases requiring hypertonic saline intervention, suggesting that the precise measurement and dynamic early warning mechanism effectively prevented the progression of risk to serious consequences.^[19]^

In terms of surgical efficiency and postoperative recovery, the intervention group also demonstrated multiple benefits. The reduction in operative time can be explained from several perspectives: temperature-controlled irrigation maintains lens clarity, stable low pressure provides a clear surgical field, and dedicated nurse management frees up team attention.^[20]^ The shortening of operative time itself holds independent clinical value, further reducing total irrigation fluid volume and absorption volume, forming a virtuous cycle. The shorter postoperative hospital stay in the intervention group reflects the combined benefits of reduced complications and improved surgical efficiency, aligning with the principles of enhanced recovery after surgery.^[21]^

Subgroup analysis revealed the robustness of the nurse-led management model. Regardless of operative duration, the intervention group effectively controlled intrarenal pelvic pressure and irrigation fluid absorption volume. In high-risk patients with an operative duration >90 minutes, although the small sample size resulted in some clinical outcomes not reaching statistical significance, the observed trends toward benefit were more pronounced – the postoperative high fever rate decreased from 35.7% to 20.0%, and hyponatremia decreased from 28.6% to 10.0%, representing absolute risk reductions exceeding 15 percentage points. This suggests that nurse-led management holds even greater value in high-risk patients and should be considered a routine safety measure for complex surgeries.^[22]^

In summary, the positive effects of the nurse-led management protocol stem from a triple mechanism: at the physical level, active pressure control severs the driving force of risk; at the monitoring level, precise measurement enables dynamic risk identification; at the organizational level, dedicated management ensures reliable execution across all steps.^[22]^ This transformation upgrades irrigation fluid management from passive coordination to active prevention and control, reflecting the deepening expansion of the nursing role towards that of a “process manager” and “safety guardian.”

This study has several limitations. As a single-center retrospective study, the potential influence of unmeasured confounding factors cannot be completely ruled out. Intrarenal pelvic pressure monitoring in the control group was not systematic, potentially introducing information bias. The irrigation fluid absorption volume was calculated based on the difference between input and output, which is less accurate than tracer methods. The sample size for the high-risk patient subgroup was small, limiting statistical power. A health economic evaluation was not conducted.

In conclusion, the operating room nurse-coordinated irrigation-fluid safety management protocol can effectively reduce intrarenal pelvic pressure and irrigation fluid absorption during URS, thereby significantly lowering the risks of postoperative infection and hyponatremia, while shortening operative time and postoperative hospital stay. This protocol transforms irrigation fluid management from an empirical model into a standardized process, reflecting the core value of the nursing profession in ensuring patient safety. It is recommended for clinical promotion and application, particularly for high-risk patients. Future multicenter prospective studies can be conducted to further validate these findings and explore their health economic value.

## Author contributions

**Conceptualization:** Qingquan Li, Hongwei Liu, Huamei Zhu.

**Data curation:** Qingquan Li, Hongwei Liu, Huamei Zhu.

**Formal analysis:** Qingquan Li, Hongwei Liu, Huamei Zhu.

**Funding acquisition:** Huamei Zhu.

**Investigation:** Huamei Zhu.

**Writing – original draft:** Huamei Zhu.

**Writing – review & editing:** Huamei Zhu.
